# Capgras Syndrome Triggered by Marital Separation: A Rare Case of Trauma-Induced Delusional Misidentification

**DOI:** 10.7759/cureus.100135

**Published:** 2025-12-26

**Authors:** Ali Z Ansari, Fizza Ahmed, Pho Q Doan, Abdul Ahad Siddiqi, Sahar Hafeez

**Affiliations:** 1 Family Medicine, William Carey University College of Osteopathic Medicine, Hattiesburg, USA; 2 Psychiatry, Merit Health Wesley, Hattiesburg, USA; 3 Neurology, Lake Erie College of Osteopathic Medicine, Bradenton, USA; 4 Internal Medicine, William Carey University College of Osteopathic Medicine, Hattiesburg, USA; 5 Pathology and Laboratory Medicine, William Carey University College of Osteopathic Medicine, Hattiesburg, USA

**Keywords:** adult-onset delusion, capgras syndrome, delusional disorder, emotional trauma, facial recognition, marital separation, psychosocial stress, risperidone, somatic delusion, trauma-focused psychotherapy

## Abstract

Capgras syndrome is a rare psychiatric condition classified under delusional misidentification syndromes, characterized by the belief that a familiar person has been replaced by an identical impostor. Although it is most commonly observed in association with schizophrenia, neurodegenerative diseases, or structural brain lesions, it may also arise in the context of acute psychological trauma or severe emotional disturbance.

We present the case of a 46-year-old African American woman with no prior psychiatric or neurological history who presented to an outpatient psychiatry clinic accompanied by her sister, with a sudden onset of delusional misidentification involving her husband and adolescent daughter. The symptoms developed shortly after the unexpected discovery of her husband’s extramarital affair and his decision to initiate divorce proceedings, events which she described as profoundly destabilizing. Within days, she became convinced that both her husband and daughter had been replaced by impostors who were physically identical but emotionally unfamiliar, leading to avoidance behaviors, interpersonal conflict, and significant functional impairment. The patient denied hallucinations or other psychotic symptoms, and cognitive screening with the Montreal Cognitive Assessment (MoCA) was within normal limits (28/30). A comprehensive medical and neurological evaluation, including laboratory workup and bedside neurological examination, revealed no abnormalities, and although neuroimaging and electroencephalography (EEG) were declined by the patient, there were no clinical indications of organic pathology. The clinical picture was most consistent with delusional disorder with features of Capgras syndrome in the context of acute psychological trauma. Treatment was initiated with low-dose risperidone, gradually titrated to 2 milligrams per day, alongside trauma-focused psychotherapy focused on narrative reconstruction and emotional integration. Over a three-month period, the patient exhibited marked improvement in insight and reduction in the intensity of her delusional beliefs, eventually acknowledging that her perceptions may have been distorted by emotional distress.

This case demonstrates a rare but clinically important presentation of Capgras syndrome triggered by acute psychosocial trauma in the absence of psychosis or neurological disease. It highlights how severe emotional stress can lead to a functional disconnect between facial recognition and emotional familiarity, resulting in delusional misidentification even in high-functioning individuals. Prompt psychiatric treatment combining antipsychotic medication and trauma-focused psychotherapy can support recovery and help prevent further psychological decline.

## Introduction

Capgras syndrome is a rare psychiatric condition categorized within the broader class of delusional misidentification syndromes. It is characterized by the persistent belief that a familiar individual, most often a close relative or household member, has been replaced by an identical-looking impostor [1]. This delusion is considered non-bizarre in structure but deeply distressing and impairing in its consequences, frequently resulting in interpersonal dysfunction and safety concerns [2]. While relatively uncommon, Capgras syndrome represents an important clinical entity, not only due to its unique symptomatology but also because it often integrates aspects of psychiatric, neurologic, and cognitive disciplines [3]. Accurate recognition is essential, as affected individuals may present without overt psychosis yet harbor delusional beliefs that significantly compromise their functioning and insight. Traditionally, Capgras syndrome has been associated with primary psychotic disorders such as schizophrenia and schizoaffective disorder [4]. However, it has also been documented in patients with neurodegenerative diseases including Alzheimer's dementia and Lewy body dementia, as well as in cases of traumatic brain injury, cerebrovascular accidents, and epileptic syndromes [4,5]. It may arise transiently in the context of delirium, mania, or pharmacologic intoxication [5]. These varied associations support the prevailing view that Capgras syndrome is not a standalone diagnosis but rather a symptom arising from disruptions in cognitive-affective integration. In some instances, the syndrome has been reported in individuals with no identifiable neurologic pathology or psychiatric history, suggesting that acute environmental or emotional stressors may play a contributory role [2].

The leading neurocognitive model of Capgras syndrome posits that it arises from a disconnect between intact facial recognition and the corresponding emotional familiarity typically evoked by familiar individuals [6]. While the visual identification of a known person remains preserved, mediated by the fusiform face area, the expected affective response, often linked to amygdalar and limbic system function, is absent or blunted. This perceptual-affective mismatch is hypothesized to create a subjective sense of strangeness or incongruity, which the patient then resolves through a delusional attribution [2,3,7]. Neuroimaging studies have frequently identified abnormalities in the right frontal and temporal cortices in affected patients, particularly in regions responsible for emotion, familiarity processing, and reality monitoring [7]. Nonetheless, not all patients with Capgras delusion demonstrate structural lesions, suggesting that functional impairments, possibly reversible, may be sufficient to disrupt the integration of recognition and emotional salience. Psychological and psychodynamic perspectives further suggest that Capgras syndrome may represent a defense against intolerable emotional conflict or interpersonal trauma [8,9]. In such frameworks, the delusion functions as a mechanism to externalize distress or to disavow painful emotional realities, such as betrayal, loss, or perceived abandonment [8]. Rather than confronting these emotions directly, the individual unconsciously reorganizes their perception of others in a manner that psychologically distances them from the source of distress [9]. While these formulations remain largely theoretical, they offer valuable insight into presentations that lack clear neuroanatomical findings and instead follow major emotional upheaval.

## Case presentation

A 46-year-old African American woman with no prior psychiatric, neurological, or major medical history presented to an outpatient psychiatry clinic accompanied by her sister for evaluation of new-onset delusional misidentification. Her sister reported a sudden and dramatic behavioral change over the preceding two weeks, characterized by emotional withdrawal, pervasive anxiety, and an unshakeable conviction that her husband and 14-year-old daughter had been replaced by impostors who were physically identical but emotionally foreign. The patient described the onset as “like waking up and realizing the people I love most aren’t real anymore.” The episode followed the recent discovery of her husband’s extramarital affair and his decision to pursue divorce after more than two decades of marriage, an event she described as “the most devastating moment of my life.” She reported feeling “numb,” “disconnected,” and “like the world lost its warmth” shortly after learning of the betrayal. Within several days, these feelings evolved into a fixed belief that her husband was not her “real husband” but an identical copy “pretending to care.” The delusion gradually extended to her daughter, whom she described as “looking exactly the same but missing something in her eyes.” These beliefs led to severe interpersonal strain, avoidance behaviors, and heightened fear within the home. She began locking her bedroom door at night, refused to eat meals prepared by her husband or daughter, and spent most of her time isolated in her room, insisting that “the real ones are gone.”

The patient had no history of psychosis, mood disorder, or dissociative symptoms. She denied auditory or visual hallucinations, thought broadcasting, insertion, or passivity phenomena. She also denied persecutory, grandiose, or somatic delusions beyond the misidentification belief. There was no evidence of flight of ideas, pressured speech, or disorganized thought. Her sleep was markedly disturbed, with difficulty initiating and maintaining sleep, leading to daytime fatigue and irritability. She reported intermittent crying spells and feelings of depersonalization, describing her surroundings as “unreal” or “dreamlike,” but she denied suicidal ideation, self-injurious behavior, or intent to harm others. She denied any use of alcohol, tobacco, or recreational drugs, and she took no prescribed or over-the-counter medications. There was no family history of psychotic, bipolar, or neurodegenerative disorders. A maternal aunt had a history of unipolar depression treated successfully with a selective serotonin reuptake inhibitor (SSRI).

Socially, the patient was well-adjusted and high-functioning prior to onset. She held a graduate degree in education and had worked for over 15 years as a school administrator. She lived in a stable suburban household with her husband and daughter, maintained strong family ties with her sister and extended relatives, and had no financial or legal difficulties. Her premorbid personality was described by relatives as conscientious, dependable, and emotionally grounded. She identified as spiritual but not religious and denied prior exposure to psychotherapy or psychiatric medications. There was no reported history of head trauma, seizures, migraines, cerebrovascular events, or chronic medical conditions such as diabetes or hypertension.

The patient appeared her stated age, was neatly dressed, and maintained good hygiene. She was alert and cooperative, though visibly anxious and emotionally blunted. Psychomotor activity was normal, and eye contact was intermittent but appropriate. Speech was soft and deliberate, with normal prosody. Her mood was described as “confused and frightened,” and her affect was constricted, with intermittent tearfulness when discussing her family. Thought processes were linear and goal-directed, with no tangentiality, circumstantiality, or loosening of associations. Her thought content was notable for the fixed delusional belief that her husband and daughter had been replaced by impostors. She described an emotional disconnect rather than perceptual alteration, stating, “They look and sound the same, but I can feel they’re not my real family.” There was no evidence of hallucinations or other psychotic content. Cognition was grossly intact, with preserved orientation to time, place, and person. Immediate and recent recall were normal. Insight was markedly impaired regarding her delusional beliefs, although she retained awareness that others perceived her experiences as unusual. Judgment was similarly impaired within the context of her misidentification delusion but preserved in other domains.

A comprehensive neurological examination revealed no focal deficits. Cranial nerves II through XII were intact. Pupils were equal and reactive, extraocular movements were full, and funduscopic examination showed no papilledema or optic pallor. Motor strength was 5/5 throughout, with normal tone and bulk. Deep tendon reflexes were symmetric, and Babinski’s sign was absent bilaterally. Sensation was intact to light touch, vibration, and proprioception. Coordination was normal, and her gait was steady without ataxia or dysmetria. There were no extrapyramidal signs, tremors, or abnormal involuntary movements. Cognitive screening using the Montreal Cognitive Assessment (MoCA) yielded a score of 28/30, with mild difficulty on delayed recall but otherwise intact visuospatial, executive, and language functions. No deficits were observed on attention or abstraction tasks. The patient was offered further diagnostic testing, including electroencephalography (EEG) to evaluate for subclinical seizure activity and structural neuroimaging to assess for potential intracranial pathology; however, she declined both studies, explaining that the procedures heightened her anxiety and did not feel necessary given her belief that the issue was primarily emotional rather than medical.

Routine laboratory and metabolic screening were obtained to rule out organic causes of psychosis. Results are summarized in Table 1. All values were within normal limits, including electrolytes, renal and hepatic function tests, thyroid panel, and nutritional markers. Inflammatory markers, including erythrocyte sedimentation rate (ESR) and C-reactive protein (CRP), were within normal range. Infectious disease testing, including human immunodeficiency virus (HIV) antibody, rapid plasma reagin (RPR) for syphilis, and the hepatitis screening panel, was negative. Autoimmune screening, including antinuclear antibody (ANA), anti-double-stranded DNA (anti-dsDNA) antibody, and extractable nuclear antigen (ENA) panel, was non-reactive. Serum ammonia, fasting glucose, cortisol, and ceruloplasmin were within normal limits. Urine toxicology was negative for amphetamines, benzodiazepines, opioids, cannabinoids, and cocaine. No metabolic derangements or evidence of systemic illness were identified.

**Table 1:** Comprehensive laboratory evaluation obtained at initial presentation, including hematologic, metabolic, endocrine, infectious, autoimmune, and toxicologic studies All results were within normal reference ranges, with no abnormalities identified to suggest organic, infectious, or systemic causes of psychosis. ANA: Antinuclear antibody; CRP: C-reactive protein; dsDNA: double-stranded DNA; ESR: Erythrocyte sedimentation rate; HIV: Human immunodeficiency virus; RPR: Rapid plasma reagin

Test	Result	Reference Range / Unit
White blood cell count	6.8 × 10³/µL	4.0–11.0 × 10³/µL
Hemoglobin	13.1 g/dL	12.0–16.0 g/dL
Hematocrit	39.2%	36.0–46.0%
Platelets	250 × 10³/µL	150–400 × 10³/µL
Sodium	138 mmol/L	135–145 mmol/L
Potassium	4.1 mmol/L	3.5–5.1 mmol/L
Chloride	102 mmol/L	98–107 mmol/L
Bicarbonate	25 mmol/L	22–29 mmol/L
Blood urea nitrogen	13 mg/dL	7–20 mg/dL
Creatinine	0.78 mg/dL	0.6–1.3 mg/dL
Glucose (fasting)	92 mg/dL	70–100 mg/dL
Calcium	9.3 mg/dL	8.5–10.2 mg/dL
Aspartate aminotransferase	22 U/L	10–40 U/L
Alanine aminotransferase	18 U/L	7–56 U/L
Alkaline phosphatase	79 U/L	44–147 U/L
Total bilirubin	0.6 mg/dL	0.1–1.2 mg/dL
Albumin	4.4 g/dL	3.5–5.0 g/dL
Thyroid-stimulating hormone	2.1 µIU/mL	0.5–4.5 µIU/mL
Free thyroxine	1.1 ng/dL	0.8–1.8 ng/dL
Vitamin B_12_	612 pg/mL	200–900 pg/mL
Folate	8.6 ng/mL	>3.0 ng/mL
Vitamin D	34 ng/mL	30–100 ng/mL
Morning cortisol	13.8 µg/dL	6–23 µg/dL
Ceruloplasmin	26 mg/dL	20–35 mg/dL
ESR	9 mm/hr	0–20 mm/hr
CRP	1.1 mg/L	<3.0 mg/L
ANAs	Negative	Negative
Anti-dsDNA	Negative	Negative
HIV 1/2 antibody	Non-reactive	Non-reactive
RPR	Non-reactive	Non-reactive
Hepatitis B surface antigen	Negative	Negative
Urine toxicology	Negative	Negative

The overall clinical presentation was most consistent with delusional disorder with Capgras-type misidentification, occurring in the context of acute psychological trauma. The absence of hallucinations, formal thought disorder, and disorganization argued against a schizophrenia-spectrum illness. The acute temporal association with severe emotional stress, combined with preserved cognition and reality testing outside the delusional content, supported a trauma-related functional disturbance in cognitive-affective integration rather than a primary psychotic or neurologic disorder.

The patient was initiated on low-dose risperidone 0.5 mg nightly, gradually titrated to 2 mg daily over ten days. The dose was selected to minimize sedation and extrapyramidal risk while addressing fixed delusional intensity. She tolerated the medication well, with no reported orthostatic changes, extrapyramidal symptoms, galactorrhea, or metabolic abnormalities. Her sleep improved significantly within two weeks, and her anxiety decreased. Over the next several weeks, she reported fewer feelings of fear toward her family, though she continued to express uncertainty about their identities. Concurrently, she began trauma-focused psychotherapy with a licensed clinical psychologist. Treatment integrated elements of cognitive processing therapy (CPT) and trauma-focused cognitive behavioral therapy (TF-CBT), with an emphasis on psychoeducation, affect regulation, and graded exploration of the delusional content through a trauma-informed lens. Early sessions focused on establishing safety, grounding techniques, and cognitive restructuring of catastrophic interpretations. Direct confrontation of the delusion was intentionally avoided, allowing the patient to explore the emotional significance of her misidentification beliefs without triggering defensive resistance. Over time, therapeutic focus shifted toward narrative reconstruction of the marital betrayal and its psychological impact.

By week six of treatment, the patient began demonstrating partial insight, articulating that her perceptions “might be emotional reactions, not literal changes.” Her affect became more expressive, and her anxiety further diminished. By week eight, she no longer endorsed misidentification beliefs regarding her daughter and had resumed daily caregiving tasks, including preparing meals and assisting with schoolwork. Her delusional conviction about her husband softened, with statements reflecting emotional, rather than perceptual, estrangement (“He feels different because I can’t trust him anymore”). Her sleep normalized, appetite returned, and she re-engaged socially with her sister and close friends. At the three-month follow-up, the patient exhibited marked clinical improvement with complete remission of delusional content. She remained adherent to risperidone 2 milligrams daily and continued trauma-focused psychotherapy on a weekly basis. She had returned to work part-time and described her sense of identity as “more grounded and connected.” Family psychoeducation and conjoint sessions were introduced to address lingering relational strain and promote understanding of her prior behaviors. Her daughter and sister reported restoration of familial trust and noted significant improvement in the patient’s emotional responsiveness. The care team recommended continuing risperidone for at least three additional months with gradual tapering contingent upon sustained stability.

## Discussion

The prevailing neurobiological model of Capgras syndrome proposes that the disorder arises from a functional disconnection between the ventral visual processing stream, which mediates conscious facial recognition, and limbic circuits, particularly the amygdala, hippocampus, and related structures, which generate the emotional sense of familiarity and attachment [10]. Under normal circumstances, the ventral stream identifies a face as known, while the limbic system provides the affective confirmation that the person is indeed familiar and emotionally significant. When this integration is disrupted, the individual may still consciously recognize a familiar face at a perceptual level but fail to experience the corresponding emotional resonance. The resulting cognitive-affective mismatch produces a sense of estrangement or unfamiliarity, prompting the mind to create a delusional explanation to reconcile this incongruity, often concluding that the familiar person has been replaced by an impostor [3]. Prior neurobiological research has implicated dysfunction within the right frontal and temporal regions, particularly in the inferior frontal gyrus, temporal pole, fusiform face area, and amygdalar pathways, in the pathophysiology of Capgras syndrome [7]. These regions are believed to integrate perceptual recognition with emotional salience, allowing familiar individuals to be both cognitively and emotionally identified. Disruption within these circuits, whether due to structural damage or functional dysregulation, can decouple recognition from affective response, resulting in the characteristic misidentification seen in this disorder [10].

In the current case, the absence of clinical signs suggesting neurological impairment and the patient’s preserved cognition and orientation suggest a functional rather than structural mechanism. Acute psychological trauma, particularly experiences involving betrayal or emotional loss, has been shown in neurobiological and psychiatric studies to transiently disrupt the connectivity between the prefrontal cortex, amygdala, and anterior cingulate cortex, which are regions that mediate emotional regulation, salience processing, and reality testing [11]. Activation of the hypothalamic-pituitary-adrenal (HPA) axis during acute stress results in elevated cortisol and catecholamine release, which can alter functional coupling among these networks. Such physiological and emotional stress responses may momentarily impair the integration of perceptual and affective processing, leading to the experience of a familiar individual as emotionally unfamiliar. In this framework, the delusional belief that a loved one has been replaced represents an involuntary, maladaptive attempt to resolve the psychological conflict between recognition and emotional detachment. This proposed mechanism is consistent with existing literature describing stress-related dissociative phenomena, such as derealization and depersonalization, which also involve disruptions in the sense of familiarity and emotional connectedness [12]. Therefore, trauma-induced Capgras syndrome may not reflect an enduring psychotic disorder but rather a transient, stress-mediated dysregulation of cognitive-affective integration within an otherwise intact neural system.

From a psychodynamic standpoint, Capgras syndrome that emerges after trauma can be understood as a defensive dissociative response to overwhelming emotional conflict. The sudden loss of emotional safety following betrayal, especially within close relationships, may create intense internal tension and disrupt the individual’s sense of attachment and trust. In this context, delusional misidentification serves as a psychological defense that externalizes distress [13]. Rather than confronting painful feelings of alienation or mistrust directly, the individual projects these emotions outward by perceiving loved ones as impostors. This process allows for preservation of internal stability by distancing the self from unbearable affect. Psychodynamically, the delusion separates opposing emotions, maintaining affection for the familiar person while attributing negative feelings to the imagined impostor [9]. In this case, the belief that the husband and daughter had been replaced likely reflected emotional estrangement and the breakdown of trust that followed betrayal. From a cognitive-behavioral perspective, trauma-related Capgras syndrome may also represent a disruption in reality appraisal, where emotional distress and dissociation lead to misinterpretation of internal experiences such as detachment, numbness, or depersonalization [14]. This explanation is consistent with theories suggesting that stress-related neurochemical changes and altered dopamine signaling can cause neutral experiences to be perceived with excessive significance [15]. Together, these perspectives suggest that trauma may precipitate Capgras syndrome through a combination of dissociative defenses, emotional dysregulation, and distorted cognitive processing, producing a temporary but deeply disorienting disturbance in the recognition of familiar individuals.

The differential diagnosis of Capgras syndrome encompasses neurological, psychiatric, and functional conditions capable of producing misidentification phenomena. Neurodegenerative disorders, including Lewy body dementia, Alzheimer’s disease, and frontotemporal dementia, were considered because they often present with cognitive decline or visuoperceptual impairments that can trigger delusional misidentification [5,6]. The patient’s preserved cognition, stable orientation, and absence of progressive deficits made these etiologies unlikely. Structural or metabolic causes, such as stroke, tumors, encephalitis, seizure disorders, or metabolic encephalopathies, were also considered because they can disrupt neural circuits involved in face recognition or familiarity. Normal neurological examination, intact cranial nerves, preserved motor and sensory function, and unremarkable laboratory, metabolic, autoimmune, and toxicology results reduced the likelihood of an underlying organic condition. Primary psychotic disorders, including schizophrenia and delusional disorder, were evaluated because Capgras syndrome can manifest within these illnesses [16]. However, the absence of hallucinations, disorganization, pervasive paranoia, or longstanding psychotic features argued against a primary psychotic disorder. Mood disorders with psychotic features were similarly unlikely given the absence of affective symptoms concurrent with the delusional beliefs. The acute onset of misidentification immediately following severe emotional trauma, combined with intact cognition and reality testing outside the delusional content, supported a functional, trauma-related mechanism rather than an organic or primary psychotic etiology. This pattern aligns with prior reports of stress-induced or context-dependent Capgras syndrome in otherwise psychologically stable individuals.

The favorable clinical outcome observed in this case supports the effectiveness of a comprehensive, multimodal treatment strategy in addressing trauma-related Capgras syndrome. Pharmacologic intervention with low-dose risperidone appeared to facilitate reduction of delusional conviction and emotional distress, likely through modulation of dopaminergic activity and enhancement of cognitive control mechanisms [17]. The patient’s good tolerability and early symptomatic improvement provided an optimal foundation for subsequent psychotherapeutic engagement. The trauma-focused psychotherapy component, integrating elements of CPT and TF-CBT, played a central role in addressing the affective and cognitive consequences of betrayal and emotional loss. Early therapeutic work emphasized stabilization, grounding, and emotional regulation, while later sessions guided the patient toward insight by linking her misidentification experiences to unresolved psychological trauma. Avoidance of direct confrontation of the delusional belief during initial sessions was particularly appropriate, as it preserved therapeutic rapport and allowed for gradual cognitive restructuring. Family participation further enhanced recovery by promoting understanding, reducing stigma, and restoring interpersonal trust, especially within the parent-child relationship. The complete resolution of symptoms and sustained functional improvement within three months reinforce the conceptualization of this presentation as an stress-related functional disturbance rather than a primary psychotic disorder.

## Conclusions

This case highlights a distinctive presentation of trauma-induced Capgras syndrome manifesting in the absence of structural or neurodegenerative abnormalities, emphasizing the potential for acute psychological stress to precipitate profound disturbances in identity recognition and emotional processing. The clinical course supports the growing recognition that certain cases of delusional misidentification may represent functional and reversible disruptions in limbic-frontal connectivity rather than manifestations of primary psychotic or neurological disease. Importantly, this case reinforces the necessity of a comprehensive diagnostic approach integrating medical, neurological, and psychosocial assessment to differentiate functional Capgras syndrome from its organic counterparts. The patient’s full remission following a combination of antipsychotic therapy and trauma-focused psychotherapy demonstrates the effectiveness of early, multidisciplinary, and individualized treatment strategies. Clinicians should remain vigilant for trauma-related functional variants of delusional misidentification, as timely recognition and intervention can restore normal cognition, prevent chronicity, and deepen our understanding of the complex neuropsychiatric interface between emotion, identity, and perception.
